# Hemizygous splicing variant in *CNKSR2* results in X‐linked intellectual developmental disorder

**DOI:** 10.1002/mgg3.2389

**Published:** 2024-02-09

**Authors:** Yuting Lou, Xinglei Shi, Guofa Su, Yufan Guo, Liuyan Gao, Ye Wang, Pu Miao, Jianhua Feng

**Affiliations:** ^1^ Department of Pediatrics The Second Affiliated Hospital, School of Medicine, Zhejiang University Hangzhou China; ^2^ Department of Pediatrics Suichang Branch of the Second Affiliated Hospital School of Medicine, Zhejiang University Hangzhou China; ^3^ Department of Pediatrics Songyang Branch of the Second Affiliated Hospital School of Medicine, Zhejiang University Hangzhou China

**Keywords:** ACMG, CNKSR2, intellectual developmental, variant, X‐linked

## Abstract

**Background:**

Intellectual disability (ID) refers to a childhood‐onset neurodevelopmental disorder with a prevalence of approximately 1%–3%.

**Methods:**

We performed whole exome sequencing for the patient with ID. And the splicing variant we found was validated by minigene assay.

**Results:**

Here, we report a boy with ID caused by a variant of *CNKSR2*. His neurological examination revealed hypsarrhythmia via electroencephalography and a right temporal polar arachnoid cyst via brain magnetic resonance imaging. A novel splicing variant in the *CNKSR2* gene (NM_014927.5, c.1657+1G>A) was discovered by exome sequencing. The variant caused a 166 bp intron retention between exons 14 and 15, which was validated by a minigene assay. The variant was not reported in public databases such as gnomAD and the Exome Aggregation Consortium.

**Conclusions:**

The variant was predicted to be damaging to correct the translation of the CNKRS2 protein and was classified as likely pathogenic according to the ACMG guidelines.

## Graphical abstract

The patient presented with X‐linked ID identified a novel splicing variant in the CNKSR2 gene (c.1657+1G>A) by exome sequencing. The variant caused a 166 bp intron retention between exon 14 and 15, which was validated by Minigene assay.

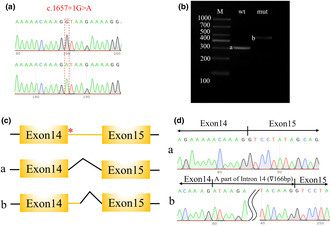

## 1 INTRODUCTION

CNKSR2, also known as connector enhancer of kinase suppressor of ras 2, is a scaffold protein that mediates mitogen‐activated protein kinase pathways downstream of Ras. Located on chromosome X, this gene contains 22 exons and consists of the Sterile Alpha Motif domain, CRIC domain, PDZ domain, Connector enhancer of kinase suppressor of ras 2/3 domain, and Pleckstrin homology domain (https://swissmodel.expasy.org/repository/uniprot/Q8WXI2).

Abnormalities in CNKSR2 cause an X‐linked intellectual developmental disorder (OMIM: #301008), which is characterized by delayed development, intellectual disability (ID), early‐onset seizures, and language delay. CNKSR2 abnormalities were first reported in 2012 in a 5‐year‐old boy with the Houge type of X‐linked syndromic intellectual developmental disorder (MRXSHG; OMIM: 301008) due to a deletion (15–21 exons) inherited from his mother, which indicated that loss of function of CNKSR2 could be pathogenic (Houge et al., 2012). Three similar hemizygous deletions incorporating CNKSR2 (Aypar et al., 2015; Vaags et al., 2014) and other types of variants have been reported (Bonardi et al., 2020; Higa et al., 2021; Vaags et al., 2014). To date, five splicing variants (c.1904+1G>A, c.2145+1G>A, c.2044+2T>A, and c.520−1G>A c.1905−2A>G) have been reported.

In this study, we report a novel alternative splicing variant of *CNKSR2* that was detected in two boys and inherited from their mother. The patients presented with seizures, ID, and developmental delay. The splicing variant c.1657+1G>A was novel, and 166 bp intron retention was verified via a minigene assay. Our results expand the genetic profile of *CNKSR2* gene variants.

## 2 METHODS

### 2.1 Ethical compliance

This study was approved by the institutional review board of the hospital (No. 1032). Informed consent was obtained from the patient's family for agreement to publish his scientific research study with unidentified individual clinical data. Clinical features, electroencephalography signals, brain magnetic resonance imaging (MRI) results, routine biochemical examination results, organ investigations, and genetic screening were also analyzed.

### 2.2 Exome sequencing

Peripheral blood samples were collected from the proband and his family. A total of 1.0 μg of genomic DNA from each sample was sheared into 200–300 bp fragments, which were subsequently analyzed with a SureSelect clinical research exome V2 (Agilent, USA) exon capture kit following the official guide. Sequencing was performed on the Illumina NovaSeq 6000 platform with a 150PE sequencing strategy.

After removing low‐quality reads and sequencing adapters, the clean data were mapped to the human reference genome (hg19) by the Burrows–Wheeler Aligner (Li & Durbin, 2009). SNPs and short insertions or deletions were analyzed by GATK. Variants were annotated with ANNOVAR (Wang et al., 2010), and those located in exonic and splicing regions with a minor allele frequency (MAF) ≤0.0001/0.005 in the SNP database (Exome Aggregation Consortium [ExAC], 1000 Genomes, gnomAD) were obtained for further analysis. The pathogenicity of the variants was evaluated according to the ACMG guidelines (Richards et al., 2015). Candidate pathogenic variants were further validated by Sanger sequencing. All the variants detected were filtered by MAF, and the variant types (exonic and splicing) are listed in Table S1.

### 2.3 Wild‐type and variant vector construction

In the general minigene assay of *CNKSR2*, the wild‐type and variant fragments were cloned and inserted into an expression vector to investigate their effect on mRNA splicing. The wild‐type target fragment was amplified by CNKSR2‐F and CNKSR2‐R, and nested PCR was subsequently performed with pECMV‐CNKSR2‐KpnI‐F and pECMV‐CNKSR2‐EcoRI‐R. The variant fragment of CNKSR2 was generated using an overlap PCR strategy. The 3′ fragment was amplified with the primer pair CNKSR2‐mut‐F and pcMINI‐CNKSR2‐EcoRI‐R. The 5′ fragment was amplified with the primer pair pcMINI‐CNKSR2‐KpnI‐F and CNKSR2‐mut‐R. The 3′ and 5′ fragments were then mixed in a 1:1 ratio and amplified with the primer pair pcMINI‐CNKSR2‐KpnI‐F and pcMINI‐CNKSR2‐EcoRI‐R. The primers used are as follows:

The wild‐type and variant fragments and the pECMV vector were digested with the EcoRI and KpnI restriction endonucleases. The digested CNKSR2 then fragments were subsequently ligated to the pECMV vector and validated via Sanger sequencing.

**Table:** 

Primer	Sequence
CNKSR2‐F	GGAGAACAGTAGGTGATGGTGGCTT
CNKSR2‐R	CCTAGCAGAAGTCTACTGAATTGGCC
pcMINI‐CNKSR2‐BamHI‐F	TCATGGGTAGGTACCCGGATCCGCTTTGTGCCCTGATTTCTCTGCC
CNKSR2‐mut‐F	AGAAGAAAAACAAAGATAAGAAAAGGAAATG
CNKSR2‐mut‐R	CATTTCCTTTTCTTATCTTTGTTTTTCTTCT
pcMINI‐CNKSR2‐EcoRI‐R	GTGCTGGATATCTGCAGAATTCCACTGAAGAAGAAGCTGGCAGC
pECMV‐CNKSR2‐KpnI‐F	TGACGATGACAAGCTTGGTACCACATTTCAGCAGTCCTCACT
pECMV‐CNKSR2‐EcoRI‐R	GTGCTGGATATCTGCAGAATTCCTCCTCATTAATATACCAATAAAGGG

### 2.4 Cell culture and transient transfection

The 293T cell line (GDC0187, CCTCC) was cultured in Dulbecco's modified Eagle's medium supplemented with 10% fetal bovine serum. Wild‐type and variant vectors were transfected into 293T cells by Lipofectamine 2000 Transfection Reagent (11668019, Invitrogen, Waltham, MA, USA) following the manufacturer's instructions.

### 2.5 RT‐PCR

Total RNA was extracted from wild‐type and variant 293T cells with an RNAiso Plus Kit (108/9109, TaKaRa). First‐strand cDNA was generated with a gDNA Eraser Kit (RR047A, TaKaRa). Wild‐type and variant fragments were amplified from pECMV‐CNKSR2‐KpnI‐F and pECMV‐CNKSR2‐EcoRI‐R, purified and investigated by Sanger sequencing. The PCR cycle condition was an initial denaturation at 98°C for 3 min; 40 cycles of 98°C 10 s, 60°C 5 min and 72°C 20 s; and a final extention at 72°C for 5 min. PCR amplification reaction system (25 μL) was 2× PrimeSTAR Max Premix 12.5 μL, primer F/R 0.5 μL (10 μM), template DNA 1 μL (100 ng/μL), and ddH_2_O 10.5 μL.

## 3 RESULTS

### 3.1 Case presentation

An 8‐year‐old boy was admitted to the hospital for epilepsy and developmental delay. He had a tonic–clonic seizure when he was 4 years old, and the symptoms included loss of consciousness, a tilted head, an enlarged eye, cyanotic lips, and stiff limbs that had persisted for approximately 1–2 min. Four months later, another two major episodes of tonic–clonic seizures started at night and lasted for 1–2 min. His head MRI showed a right temporal polar arachnoid cyst, and a dynamic electroencephalogram (2019.06.12) indicated epileptiform discharge (mainly in the left parietooccipital area). The patient was treated with sodium valproate oral solution (40 mg/mL) gradually increasing from the initial dose (80 mg, q12h) to the control dose (90 mg, q12h). Finally, the epilepsy was well controlled.

At the ages of 5 and 7 months, the focal motor seizures were triggered by fever (body temperature of 38°C), which persisted for 10 min and manifested as right upper limb shaking. The condition was controlled again with sodium valproate oral solution (100 mg, q12h).

At the age of 7, he had onset multifocal unilateral impaired awareness seizures with no obvious cause, with symptoms of sudden falling to the right side (when standing or sitting), slow blinking of the right eye, slow shaking of the right hand, and loss of consciousness. The blood concentration of sodium valproate was 43.7 μg/mL, which was lower than the normal range (ref: 50–120 μg/mL). The treatment strategy was changed to sodium valproate tablets (200 mg, tid), and the seizures were controlled again.

At the age of 8, seizures occurred again, but the current blood concentration of valproate was 58.1 μg/mL (ref: 50–120 μg/mL); therefore, the treatment strategy was not adjusted. His parents reported frequent seizures approximately 1–4 times per month.

Reviewing his development history, we found that his development was delayed, as he walked later (1.5 years old) and only mastered simple or repeat words at 4 years of age. In his family, his brother also exhibited developmental delay; he started to walk at 3 years, spoke at 5 years, and had ADHD.

### 3.2 Identification of splicing variants in *CNKSR2*

**FIGURE 1:**
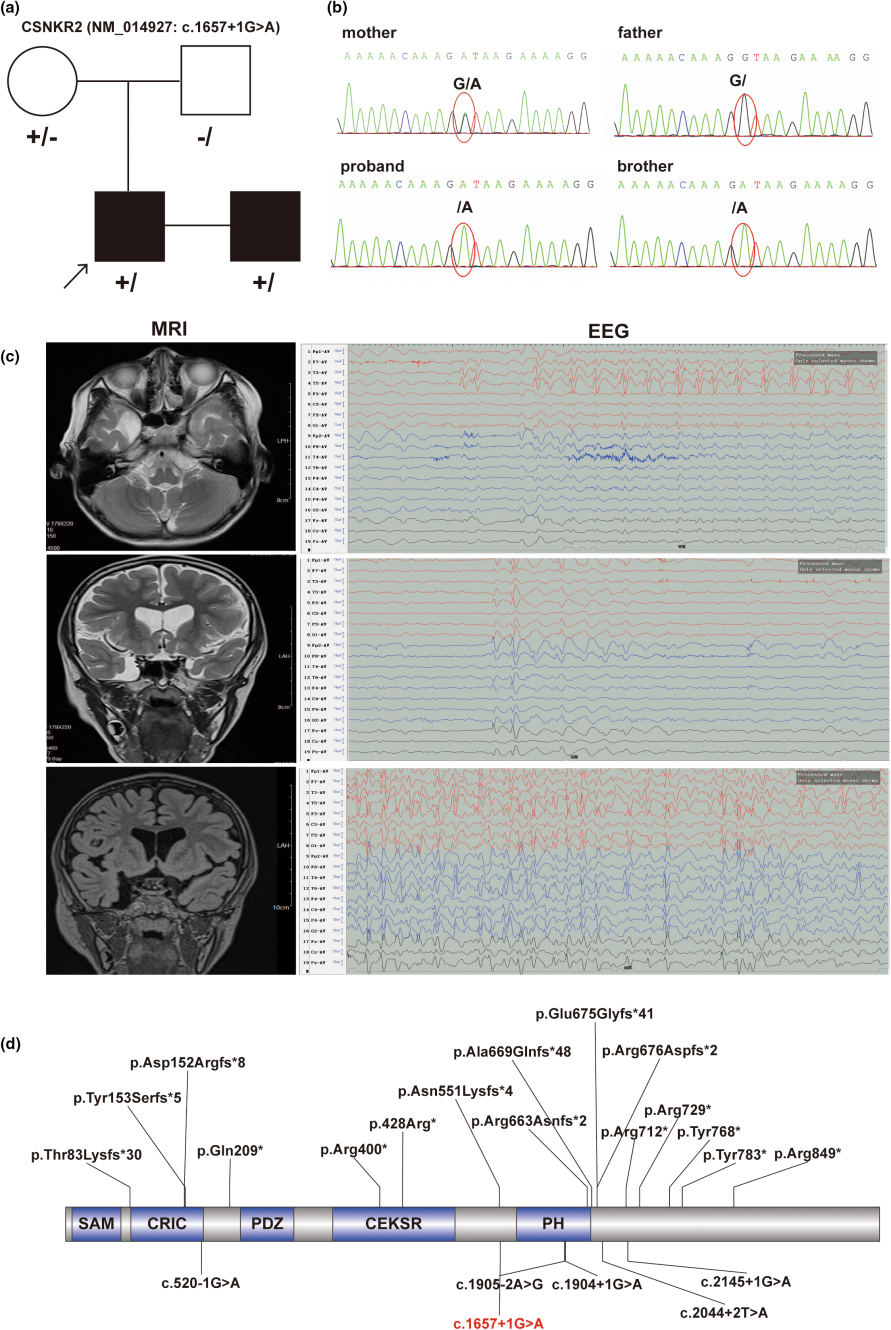
Genetic and clinical features. (a) Pedigree and genetics of CNKSR2 (NM_014927: c.1657+1G>A) in the family. +/: hemizygous variant; +/−: Heterozygous variant; −/: wild‐type variant. Black arrow: proband, black color: affected patients. (b) The genotype of *CNKSR2* (NM_014927: c.1657+1G>A) was validated by Sanger sequencing. (c) The MRI results showed a right temporal polar arachnoid cyst. (d) Summary of reported variants of CNKSR2. Black: reported variants; red: variant from this study. MRI, magnetic resonance imaging. The asterisk (*) denotes the premature termination codon.

An X‐linked splicing variant of *CNKSR2* (NM_014927.5: c.1657+1G>A) was identified by whole‐exon sequencing (the reference genome version was hg19). In addition, no candidate CNVs associated with the patient's phenotype were detected. The variant detected in our patient was inherited from his mother and was confirmed by Sanger sequencing (Figure 1a,b). His brother, who had a similar phenotype, was also detected to have the same splicing variant. The splicing variant was not observed in the gnomAD, ExAC, or ClinVar databases. Moreover, five other splicing variants of *CNKSR2* were reported (Ito & Nagata, 2022), one in the CRIC domain around the N‐terminus and the other located around the PH domain (Figure 1d). c.1657+1G>A in our patient was classified as likely pathogenic according to the ACMG guidelines. The variant in our patient was uploaded to the ClinVar database (accession number: SCV004176289).

### 3.3 Pathogenic variant validation by minigene

**FIGURE 2:**
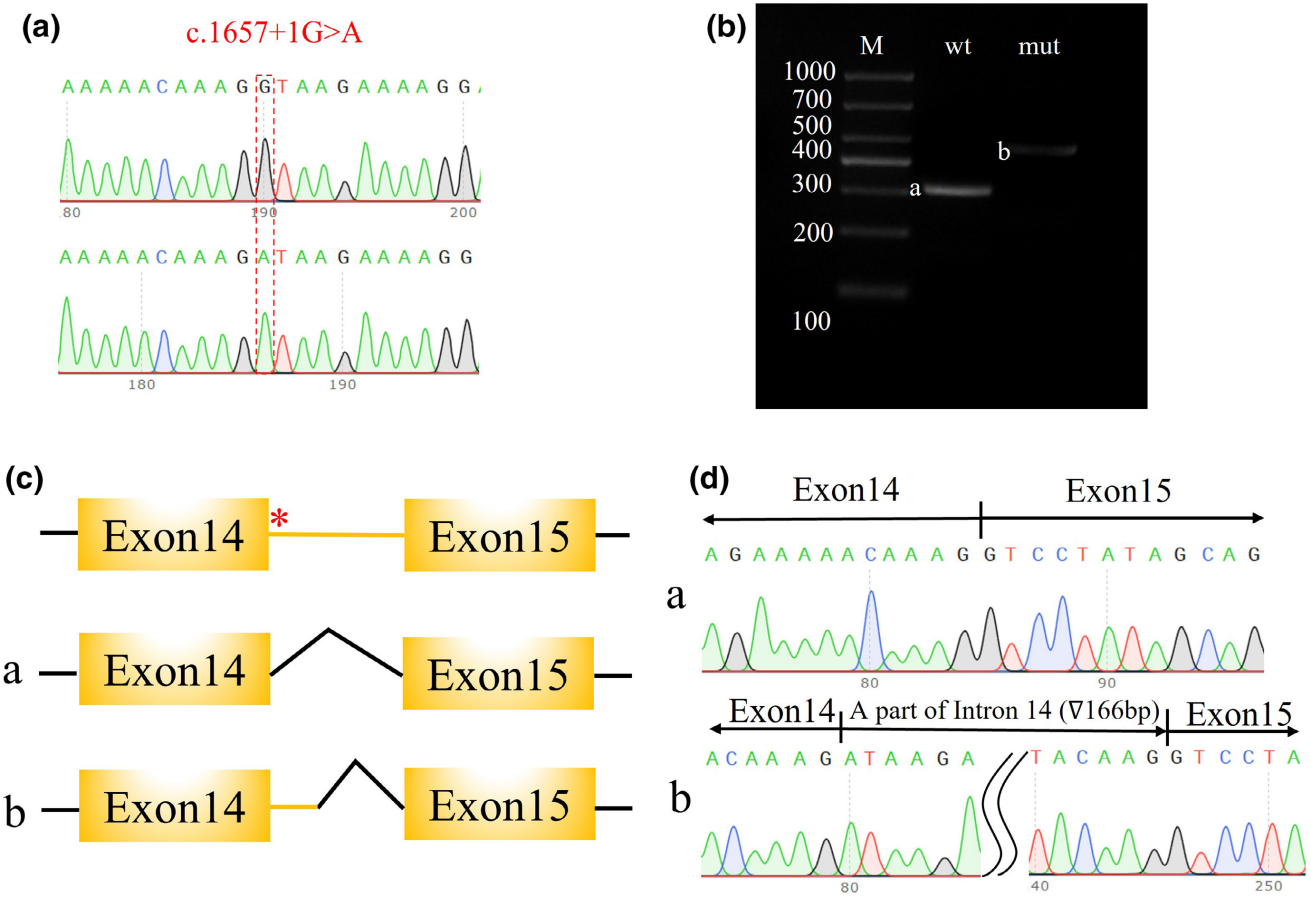
Genetic funding was obtained for our patient. (a) The wild‐type and variant (c.1657+1G>A) fragments were subsequently cloned and inserted into the pECMV vector. (b) PCR products confirmed by agarose gel electrophoresis revealed longer bands, which indicated abnormal splicing. a: wild‐type fragment; b: abnormal splicing fragment. (c and d) Abnormal and normal splicing schematic maps. (c) *: variant site. c.1657+1G>A is located behind the end of exon 14. a: Normal splicing model between exons 14 and 15. b: Abnormal splicing model influenced by the variant. (d) a: The reference sequence of the *CNKSR2* fragment from exons 14 to 15 without the c.1657+1G>A variant; b: 166 bp intron retention between exons 14 and 15 influenced by the c.1657+1G>A variant.

To further investigate the effect of the identified CNKSR2 splicing variant, a minigene assay was designed and performed. After confirming the presence of the wild‐type and variant genes in the expression vectors (Figure 2a), vector transfection, RNA isolation, reverse transcription, and target fragment amplification were performed. A larger band was observed in the variant sample, which indicated possible intron retention (Figure 2b). Sanger sequencing of the target fragments revealed a 166 bp intron retention region between exons 14 and 15 (Figure 2c,d).

## 4 DISCUSSION

The X chromosome, which constitutes approximately 5% of the human genome, is responsible for approximately 15% of the genes currently linked to ID. Advances in identifying genes associated with X‐linked intellectual disability have been made by employing high‐throughput technologies (Neri et al., 2018). CNKSR2 is highly expressed in the brain and plays a crucial role in Ras signaling‐mediated neuronal proliferation, migration, and differentiation (Lim et al., 2014). KCNSR2 is broadly expressed in the brain, including in the hippocampus, amygdala, and cerebellum (Herrero et al., 2020), possibly leading to various clinical features (Table 1). The main clinical features of these patients were ID, language defects, and epilepsy. Most of the variants were nonsense, and two patients had missense variants. There seems to be no significant association between genotype and clinical phenotype.

**TABLE 1:** Clinical features of patients with CNKSR2 variations.

Family ID	CNKSR2 variant a	Gender	Affected patients	Segregations	Intellectual disability	Epilepsy/ seizures	Hyper‐ activity	Language defect	Other clinical features	Ref. (PMID)
#1	Deletion Xp22.12 (21,375,312–21,609,484)	Male	Proband	Maternal	Mild/moderate	Yes	Yes	Yes	Borderline	22511892 25223753
#2	Deletion Xp22.12 (20,297,696–21,471,387)	Male	Proband	Maternal	Yes	Yes	Yes	Yes	Microcephaly	25223753
	Deletion Xp22.12 (20,297,696–21,471,387)	Male	Brother	Maternal	Yes	Yes	Yes	Yes		
	Deletion Xp22.12 (20,297,696–21,471,387)	Female	Mother	NR	Mild	No	NR	NR		
#3	Deletion Xp22.12 (21,193,947–21,707,169)	Male	Proband	Maternal	Yes	Yes	Yes	Yes	Non‐specific periventricular hite matter hyperintensity	25223753
	Deletion Xp22.12 (21,193,947–21,707,169)	Male	Brother	Maternal	Yes	No	Yes	Yes		
#4	Frameshift (g.21,458,832_3insA, p.D152Rfs*8)	Male	Proband	Maternal	Yes	Yes	Yes	Yes	Minor cortical atrophy	25223753
	Frameshift (g.21,458,832_3insA, p.D152Rfs*8)	Male	Brother	Maternal	Yes	Febrile	Yes	Mild		
	Frameshift (g.21,458,832_3insA, p.D152Rfs*8)	Male	Brother	Maternal	Yes	Yes	Yes	Yes		
#5	Deletion Xp22.12 (21,328,677–21,670,497)	Male	Proband	Maternal	Yes	Yes	NR	Yes		25754917
#6	Nonsense (c.2134 C>T, p.Arg712*) b	Male	Proband	Maternal	Yes	Yes	Yes	Yes		28098945
	Nonsense (c.2134C>T, p.Arg712*) b	Male	Brother	Maternal	Mild	Yes	Yes	Mild		
	Nonsense (c.2134C>T, p.Arg712*) b	Female	Sister	Maternal	Mild	Mild	NR	Mild		
	Nonsense (c.2134C>T, p.Arg712*) b	Female	Mother	NR	No	Febrile	NR	No		
#7	Nonsense (c.2185C>T, p.Arg729*)	Male	Proband	De novo	Yes	Yes	Yes	Yes	Autism performance	30397616
#8	Nonsense (c.2304G>A, p.Trp768*)	Female	Proband	De novo	Mild	Yes	No	No		31414730
#9	Deletion Xp22.12 (21,606,698–21,616,207)	Male	Proband	Maternal	Yes	Yes	Yes	Yes	White matter lesions	32245427
	Deletion Xp22.12 (21,606,698–21,616,207)	Male	Brother	Maternal	Yes	Yes	Yes	Yes	Small multifocal white matter lesions	
	Deletion Xp22.12 (21,606,698–21,616,207)	Female	Mother	De novo	Mild	Febrile	No	Mild		
#10	Frameshift (c.2024_2027delAGAG, p.Glu675Glyfs*41)	Male	Proband	De novo	Mild	Yes	NR	Yes		32197126
#11	Frameshift (c.246–247delAG, p.Thr83Lysfs*30)	Male	Proband	De novo	Mild	Yes	Yes	NR		32197126
#12	Frameshift (c.457_461del, p.Tyr153Serfs*5)	Male	Proband	Maternal	Moderate/severe	Yes	NR	Yes		32197126
#13	Deletion Xp22.12 (21,523,673–21,558,329)	Female	Proband	NR	Mild	Yes	Yes	Yes		32197126
#14	Deletion Xp22.12 (21,609,392–21,619,786)	Male	Proband	De novo	Moderate	Yes	Yes	Yes		32197126
#15	SPLICING (c.1904+1G>A)	Male	Proband	Maternal	Mild	No	Yes	No		33298018
#16	Nonsense (c.625C>T, p.Gln209*)	Male	Proband	Maternal	Yes	Yes	Yes	Yes		34114993
#17	Nonsense (c.2349T>G, p.Tyr783*)	Male	Proband	De novo	Yes	Yes	Yes	Yes	Autism	34266427
#18	Missense (c.1537C>T, p.Pro513Ser)	Male	Proband	Maternal	Yes	Yes	No	Yes		
#19	Frameshift (c.1988_1989del, p.Arg663Asnfs*2)	Male	Proband	De novo	Yes	Yes	Yes	Yes		
#20	Frameshift (c.1653_1656del, p.Asn551Lysfs*4)	Male	Proband	De novo	Yes	Yes	No	Yes		
#21	Nonsense (c.2545C>T, p.Arg849*)	Male	Proband	Maternal	Yes	Yes	Yes	Yes		
#22	Splicing (c.2145+1G>A)	Male	Proband	De novo	Yes	Yes	Yes	Yes		
#23	Deletion Xp22.12 (21,278,397–21,678,707)	Male	Proband	Maternal	Yes	Yes	Yes	Yes	Autism	
#24	Nonsense (c.1198C>T, p.Arg400*)	Male	Proband	De novo	Yes	Yes	Yes	Yes		
#25	Splicing (c.2044+2T>A)	Male	Proband	De novo	Yes	Yes	Yes	Yes		
#26	Splicing (c.520‐1G>A)	Male	Proband	De novo	Yes	Yes	Yes	Yes		
#27	Frameshift (c.2005del, p.Ala669Glnfs*48)	Male	Proband	De novo	Yes	Yes	No	Yes		
#28	Splicing (c.1905‐2A>G)	Male	Proband	De novo	Yes	Yes	No	Yes		
#29	Frameshift (c.2026_2027del, p.Arg676Aspfs*2)	Male	Proband	De novo	Yes	Yes	Yes	Yes	Autism	
30#	Nonsense c.1282C>T (p. Arg428*)	Male	Proband	Maternal	Severe	No	No	Yes	Autism macrocephaly hydrocephalus	36105777
	Nonsense c.1282C>T (p. Arg428*)	Female	Mother	NR	NR	No	No	NR		
	Nonsense c.1282C>T (p. Arg428*)	Female	Sister	Maternal	NR	NR	No	NR		
#31	Missense (c.2740A>G; p.Arg914Gly)	Male	Proband	Maternal	Yes	Yes	Yes	Yes		37929438
#32	Splicing (c.1657+1G>A)	Male	Proband	Maternal	Yes	Yes	Yes	Yes		Our patient
	Splicing (c.1657+1G>A)	Male	Brother	Maternal	Yes	No	Yes	Yes		

Abbreviation: ID, intellectual disability.

RNA splicing refers to the crucial process of intron removal from pre‐mRNAs and exons joining together to form the mature transcript of the gene. By skipping or retaining exons or introns, several isoforms can be generated from the same gene. Variants at splicing sites result in either exon skipping or intron retention, which results in an abnormal coding sequence and altered length of the protein (Matera & Wang, 2014), while others may create novel isoforms with altered or even gain‐of‐function properties (Kelemen et al., 2013). Abnormal splicing in neurodevelopmental diseases was also reported to influence the gene expression level, protein maturation, neuronal development, and disease pathology (Biamonti & Caceres, 2009). For example, variants of splicing factors were reported to be associated with autism spectrum disorder (ASD) and synaptic function for both ASD and ID (Irimia et al., 2014; Parikshak et al., 2016). Here, we found a novel splicing variant in the *CNKSR2* gene (NM_014927: c.1657+1G>A). The mutation was maternal and was verified by Sanger sequencing. The impact of this splicing variant was shown in the minigene assay, in which a 166 bp intron was retained between exons 14 and 15. This may have affected the protein level of CNKSR2 and was related to the developmental delay of our patients.

To date, splicing variants in CNKSR2 have been rare, and only five have been found in previous reports. The clinical features of these patients were heterogeneous. The first reported patient with a splicing variant presented a mild phenotype, and there was no language disorder or seizures (Zhang et al., 2020). As more variants were discovered, patients with other splicing variants continued to exhibit different phenotypes, with ID, language disorders, seizures, and ADHD (Higa et al., 2021). The novel c.1657+1G>A variant was detected in our patient; he presented with developmental delay and seizures. In terms of language, he can speak only simple overlapping words. The epilepsy in our patient was first controlled with medication but has since become refractory to treatment and is no longer controlled.

In summary, our study expands the *CNKSR2* gene splice variant spectrum. A minigene assay verified the effects of the variation. However, further functional experiments are needed to determine the causes of these variations and to clarify the association between CNKSR2 and ID.

## AUTHOR CONTRIBUTIONS

YL and SQX designed and organized the study. YL, LQC, and TFS acquired the clinical data, prepared the samples from the family members, and interpreted the genetic analyses. JYF did literature searching. JYF and YL wrote the manuscript that was edited by all other authors. JHF reviewed and edited the final version of the manuscript. All authors read and approved the final version of the manuscript.

## CONFLICT OF INTEREST STATEMENT

The authors declare that there are no conflicts of interest.

## Supporting information


Table S1:

## Data Availability

The data that support the findings of this study are available from the corresponding author upon reasonable request.
